# Monocytes and Macrophages as Viral Targets and Reservoirs

**DOI:** 10.3390/ijms19092821

**Published:** 2018-09-18

**Authors:** Ekaterina Nikitina, Irina Larionova, Evgeniy Choinzonov, Julia Kzhyshkowska

**Affiliations:** 1 Department of Episomal-Persistent DNA in Cancer- and Chronic Diseases, German Cancer Research Center, 69120 Heidelberg, Germany; 2 Department of Oncovirology, Cancer Research Institute, Tomsk National Research Medical Center, Russian Academy of Sciences, Tomsk 634050, Russia; 3 Department of Translational Cellular and Molecular Biomedicine, Tomsk State University, Tomsk 634050, Russia; mitrof_@mail.ru (I.L.); Julia.kzhyshkowska@medma.uni-heidelberg.de (J.K.); 4 Department of Molecular Oncology and Immunology, Cancer Research Institute, Tomsk National Research Medical Center, Russian Academy of Sciences, Tomsk 634050, Russia; 5 Head and Neck Department, Cancer Research Institute, Tomsk National Research Medical Center, Russian Academy of Sciences, Tomsk 634050, Russia; choynzonov@tnimc.ru; 6 Institute of Transfusion Medicine and Immunology, Medical Faculty Mannheim, Heidelberg University, 68167 Heidelberg, Germany

**Keywords:** monocyte/macrophage, virus, persistence, reservoir, cell response, inflammation, cancer

## Abstract

Viruses manipulate cell biology to utilize monocytes/macrophages as vessels for dissemination, long-term persistence within tissues and virus replication. Viruses enter cells through endocytosis, phagocytosis, macropinocytosis or membrane fusion. These processes play important roles in the mechanisms contributing to the pathogenesis of these agents and in establishing viral genome persistence and latency. Upon viral infection, monocytes respond with an elevated expression of proinflammatory signalling molecules and antiviral responses, as is shown in the case of the influenza, Chikungunya, human herpes and Zika viruses. Human immunodeficiency virus initiates acute inflammation on site during the early stages of infection but there is a shift of M1 to M2 at the later stages of infection. Cytomegalovirus creates a balance between pro- and anti-inflammatory processes by inducing a specific phenotype within the M1/M2 continuum. Despite facilitating inflammation, infected macrophages generally display abolished apoptosis and restricted cytopathic effect, which sustains the virus production. The majority of viruses discussed in this review employ monocytes/macrophages as a repository but certain viruses use these cells for productive replication. This review focuses on viral adaptations to enter monocytes/macrophages, immune escape, reprogramming of infected cells and the response of the host cells.

## 1. Introduction

Infectious agents must find, attack and enter permissive cells, avoid the immune response and productively replicate their genomes. Additionally, these agents must consolidate, that is, establish a persistent infection in an organism with the aim of viral army replenishment and a continuation of its lifecycle. To be a viral reservoir, cells must meet the following characteristics: have a sufficient lifespan, be able to avoid apoptosis and the immune response and have facile interactions with other cell populations. Some cells have these characteristics inherently but others can be transformed and adapted by viral infection, resulting in similar effects. These findings are validated by several results described in this review. Monocytes (Mo) by definition are non-dividing cells with a short half-life that makes viral replication difficult or almost impossible. A limited lifespan, limited cell resources, programmed cell death and an immune response are major restrictive characteristics of monocytes/macrophages as a permissive system and/or a reservoir. Nevertheless, monocytes/macrophages have several appealing characteristics as a target for viral infection, thus viruses have found ways to avoid the limitations and adapt these cells for their replication. Monocytes are broadly represented in the blood stream; they continuously differentiate from bone marrow precursors and enter into the circulation where they can be exposed to viral particles. Monocytes are at the front line in the defence against foreign invasion by microorganisms, providing the first virus-cell contact upon infection. Monocytes are professional antigen-presenting cells with a broad repertoire of receptors on the cell surface and high phagocytic activity, which can be exploited by viruses. As a host defender, upon infection, monocytes change their cytokine/chemokine pattern, which directs cell differentiation into long-lived macrophages (Mφ) and promotes migration into tissue where they become infected resident cells. This gives viruses the possibility to disseminate through all organs and tissues, including the brain (also known as the “Trojan horse” hypothesis [1,2]), forming stable and long-lived reservoirs which can be used for virus replenishment upon favourable conditions for reactivation. Additionally, Mo/Mφ are involved in the vertical transmission of virus from an infected mother to the developing foetus in utero [3]. Mo/Mφ can actively interact with other cell populations through direct cell-to-cell contacts, providing a basis for virus dissemination. Finally, monocytes/macrophages are powerful producers of cytokines/chemokines that are involved in the immune response and inflammation initiation and this can be modulated and utilized by viruses for infection spread and establishment. In the current review, these aspects are discussed in detail and are presented with representative examples.

Many types of viruses have been subject to comprehensive investigations exploring monocyte/macrophages-virus interactions, the molecular basis of these interactions, response and permissiveness. For some viral families such as *Retroviridae* (e.g., human immunodeficiency virus or HIV) and *Herpesviridae* (e.g., cytomegalovirus or CMV), information has been obtained and presented in detail due to their great importance to public health. This is highlighted by their worldwide role in millions of deaths each year. However, several questions remain unanswered due to the versatile biology of viruses, their resourcefulness and the diversity of monocyte/macrophage polarization and tissue specificity.

This review analyses previously published data on the myeloid cell lineage, with a primary focus on the interaction between monocytes/macrophages and viruses. This paper intends to highlight the aspects of cell regulation that viruses adopt to enable viral persistence, beginning with viral entry and then examining the launching of vital pathways and the regulation of many processes. Intriguing data presented in the literature has shown that, despite their small sizes and genomes, viruses can be very powerful machines responsible for specific changes in cell regulation and function. Certain viruses that seek monocytes/macrophages frequently possess the ability to change cellular decisions regarding fate and decoy apoptosis and can alter morphology and polarize cells. These abilities, therefore, lead to cytokine/chemokine expression modification and escape from the immune system. The basic biology of viral reservoirs, knowledge of viral transmission systems and “backup” cells will be of great help in the advancement of effective viral therapies, allowing the development of potential life-saving techniques. Recent achievements in this area of investigation are also summarized in this review.

## 2. Monocytes and Tissue Macrophages

Macrophages are key cells of the immune system that orchestrate various physiological and pathological processes of an infectious and non-infectious nature. Macrophages were discovered by Russian zoologist Élie Metchnikoff in the late nineteenth century [4]. Initial discovery focused on the phagocytic activity of macrophages, which is important for host defence against infection and for maintenance of ‘housekeeping’ functions such as the removal of apoptotic cells and remodelling of the extracellular matrix [5]. Accumulating data has revealed that macrophages play a valuable role in systemic metabolism, cold adaptation, tissue homeostasis and development, the pathology of chronic inflammation, cancer, cardio-metabolic disorders and neurodegeneration [6].

All macrophages take various forms (with various names) throughout the body and are designated as histiocytes, Kupffer cells, Hofbauer cells, alveolar macrophages and microglia, among others. Despite heterogeneity, tissue-resident macrophages are derived from three sources: yolk sac, foetal liver and hematopoietic stem cells in the bone marrow [7].

Major biological activities of macrophage include phagocytosis, antigen presentation and the release of cytokine (pro-inflammatory/anti-inflammatory mediators), antibacterial substances and enzymes that remodel the extracellular matrix [8]. Macrophages attract and activate other cells of the adaptive immune system, in particular T cells, to sites of chronic inflammation. Further, macrophages are able to sense the time at which an injury is terminated and thus start the resolution process of inflammation and the control of the healing phase [9].

Depending on the timeframe and tissue, several intrinsic, extrinsic and tissue-environmental stimuli promote monocyte polarization. Specific stimuli include cytokines, growth factors, prostaglandins, fatty acids and pathogen-derived molecules [10]. Mφ phenotypes represent a wide spectrum of activation states which are not restricted to the classical M1 (classically IFN-γ-activated) and M2 (alternatively IL-4-activated) subtypes [9]. M1-like polarized macrophages are characterized by a high level of phagocytic activity and an elevated secretion of proinflammatory cytokines and chemokines, which induces Th1 response activation and facilitates complement-mediated phagocytosis and type I inflammation. M1-like polarized macrophages also carry out phagocytosis of microorganism and matrix debris in the early phases of healing and have high antigen presentation capacity [10]. In several infections, M1 polarization favours virus establishment, as shown for HIV-1, which induces acute inflammation and promotes the recruitment of monocytes and T cells on site [11]. M2 macrophages comprise a wide range of macrophage subtypes, including tumour-associated macrophages, healing macrophages and macrophages found in chronic inflammatory conditions; thus, they play a crucial role in carcinogenesis and inflammation-dependent diseases (e.g., neurodegenerative disorders) [12]. M2-like macrophages modulate the Th2 response by producing anti-inflammatory mediators, leading to neutrophil, monocyte and T lymphocyte recruitment; they are highly endocytic and partially phagocytic; they are involved in a variety of functions including repair mechanisms, homeostasis, metabolic processes and pathogenesis. Macrophages possess high plasticity. Data from several studies has demonstrated that macrophages switch their polarization upon changes in the micro environmental conditions from M1 to M2 and vice versa [10].

Both acute and chronic inflammatory programming of macrophages can be utilized by viruses for their dissemination, replication and survival. M1-polarized cells are susceptible to viruses and they recruit other cell populations to the inflammation site, which favours virus transmission and dissemination. By contrast, M2 macrophages are involved in chronic disease and ensure permissiveness and the tissue distribution of viruses, forming a life-long reservoir of infection able to be activated and replenished upon conducive conditions.

## 3. Viruses Infect Monocytes and Macrophages

**Figure 1:**
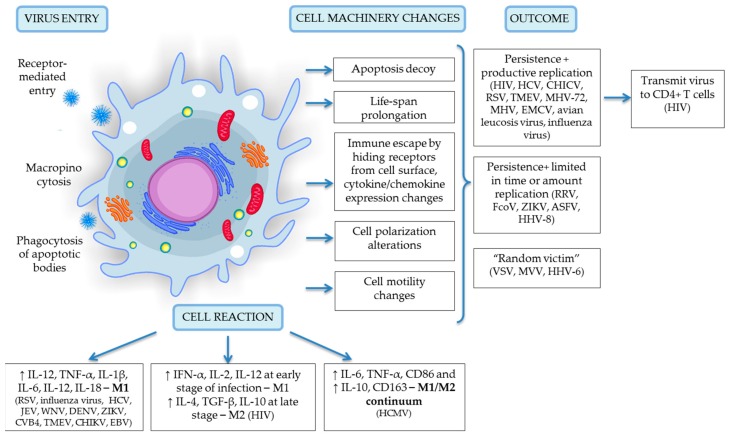
A schematic representation of virus entry, cell reaction and machinery changes and viral outcome upon infection specific for the monocyte/macrophage system. Details of depicted interactions are discussed in detail in the text above.

The first reports on the topic of myeloid cell response and reaction to viruses date back to 1978 [13,14]. The questions of permissiveness and the potential of monocytes and macrophages to be a reservoir for infectious agents was and still is, very intriguing to scientists. Knowledge in this area is interesting because it can lead to the basic understanding of many processes, such as virus-cell interactions that are receptor-based, cell-to-cell transmission upon infection, persistence, genetic changes of cell machinery and therapy applications to certain cohorts of patients. Circulating monocytes and macrophages play one of the most important roles in the protection of the organism against viral infection. Through use of animal models and viruses associated with lethal disease, researchers have made the surprising discovery that these cells can be defined as part of a permissive system, carrying special “parcels” that assist viral spread into susceptible sites (e.g., the central nervous system) and virus dissemination to other cells [14,15,16]. Later experiments on human infection (tissue-derived cells, post-mortem tissue samples, human cell cultures) revealed many spectacular functions of monocytes/macrophages and the diverse adaptations of viruses to this particular cell system [17,18,19,20]. Today, an important role of monocytes/macrophages has been shown for the persistence or spread of more than 35 viruses belonging to 13 different families. Among them are ssRNA and dsDNA agents, which lead to a variety of diseases including formidable immunodeficiency syndrome, virus-induced microcephaly and Guillain–Barré syndrome [3,18]. Types of viruses, species affected, models and organisms used for experiments, monocyte/macrophage reaction and cell polarization changes upon infection and other valuable information are summarized in Table 1 and depicted in Figure 1. These data concern humans [17,19,21,22], mammals in general [23,24] and amphibian species [25].

## 4. Viral Entry into Monocytes and Macrophages

The initial step of infection is virus contact with the host cell and introduction of its material into the cell. The viral capsid is usually neutrally charged and viruses and cells do not naturally attract each other; therefore, the virus must find a way to move near to a host cell. Most viruses do this by attachment to a susceptible cell that contains a receptor. Initial virus-cell interaction can occur through low- or high-affinity receptors, leading to the activation of many signalling pathways, which in turn can provide virus escape from immune reaction, prevent host cell apoptosis or adapt the cell to the permissiveness. After initial contact with the cell surface, viruses can penetrate into the cell through endocytosis, phagocytosis, macropinocytosis or membrane fusion. It can be assumed that the uptake pathway and type of receptors involved are the first adaptation step necessary to the prosperous survival of viruses.

Endocytosis is a form of a clathrin-mediated molecule transportation. As described in detail by Grove J. and Marsh M. (2011) [72], the receptor-mediated interaction can be represented as follows: receptors can target viruses for endocytosis; receptors may be used to activate specific signalling pathways leading to virus entry; receptors may directly drive fusion/penetration events, either at the surface of a target cell or within endocytic compartments, that can be achieved by inducing conformational changes in key virus surface structures. Phagocytosis is a type of endocytosis that involves the actin-dependent formation of vesicles. Macropinocytosis is an endocytic process of nonselective uptake of extracellular fluid, which can be induced upon activation of growth factor signalling pathways and can also be exploited by several viruses [73]. Virus surface components and the composition of viral coats determine virus-cell interactions and orchestrate their attachment to the cell surface. For example, enveloped viruses, which are packed in a membrane highly similar to that of the host cell, can fuse directly at the plasma membrane after interaction with cell surface receptors [72], as has been shown for HIV-1 [55]. The various mechanisms given here are widely exploited by viruses against many cell populations, in addition to monocyte/macrophage cells.

Monocytes/macrophages are known to be a professional antigen-presenting cells and “professional” phagocytes. These cells are well equipped with general receptors and several sensors, namely, pattern recognition receptors. These receptors can initiate and control immune responses to invading pathogens and maintain tolerance to self-antigens [74]. Data representing virus-cell binding events for viruses that cause lethal or clinically severe diseases are summarized in Table 2. Receptors and proteins involved in virus-host interactions define the next steps of the viral life cycle. For receptor-mediated entry, viruses can employ both nonspecific receptors, where a virus accesses a broad range of cell populations, or highly specific interactions between the virus and cell surface receptors, where a virus infects a limited set of target cells; this determines the tropism of viral infection. Several viruses use classical receptors and transmembrane proteins that are widely represented in cells and are not restricted to the monocyte/macrophage population, such as nucleolin by the respiratory syncytial virus [75]; sialic acid sugars by the influenza virus [76], mouse hepatitis virus [77] and Theiler’s murine encephalomyelitis virus [78]; and phosphatidylserine by the vesicular stomatitis virus [79]. This strategy provides effective interactions with the cell and allows viruses to infect a wide range of cells. Despite the low-affinity of these interactions, further cellular reactions and cascade activation could provide an advantage to viruses in survival. For example, in chronic lymphocytic leukaemia, the nucleolin activation in cells result in stabilization of Bcl-2 mRNA, with subsequent overproduction of Bcl-2 protein and avoidance of apoptosis [80]. Sialic acids are highly conserved and abundant in large numbers in virtually all cells, which makes them a good target for viruses. RSV uses the hemagglutinin glycoproteins on their surface to directly bind to the sialic acids of erythrocytes as a first step of virus interaction with the host that helps to avoid immune response and enables spreading within the organism. Several viruses (HIV, CMV, RSV, KHSV) use heparan sulphate proteoglycans, which have been considered to be a nonspecific cell surface receptor based on their interactions with the positive motifs of viral proteins [11,81,82]. However, heparan sulphate proteoglycans play multiple roles in assisting viral infection. It has been suggested that heparan sulphate could serve as a specific receptor for viral infection [83]. Heparan sulphate shows activity in assisting both viral binding and viral entry. In the case of HIV infection, these receptors facilitate the internalization of viral trans activator protein, Tat and increase the levels of cytokines, promoting cell proliferation to increase the incidence of cancer and neurotoxicity in the central nervous system. The Tat protein might spread into the uninfected cells to cause the non-permissive cells to be susceptible to HIV infection [83]. Phosphatidylserine plays a valuable role in cell cycle signalling, specifically in relationship to apoptosis. In the viral membrane, phosphatidylserine mimics the membrane fragments generated during cellular apoptosis, which leads to macrophage recognition and internalization by macropinocytosis [73], which then leads to its infection and enables immune escape.

Other viruses bind to specific cofactors: FcγRII and FcγRIII and occludin for coxsackieviruses [42,73]; CD4 and coreceptors CXCR4/CCR5 or human mannose receptor C-type 1 (hMRC1) for human immunodeficiency virus [11,86]; and β1/β3 integrins and TLR3/TLR9 for cytomegalovirus [82]. Redundancy and conservation of receptors determines viral tropism, spread and pathogenesis of disease [90]. Binding to FcγRII and FcγRIII receptors, coxsackievirus launches signalling pathways resulting in the induction of both tumour necrosis factor-α and interleukin-1α production by human macrophages [91], which are involved in inflammation onset Occludin plays an important role in coxsackievirus B (CVB) entry by launching macropinocytosis and occludin also plays a role in avoidance of immune surveillance [73]. Occludin may serve as a scaffold to recruit and anchor signalling or regulatory molecules, such as a caveolin, in the vicinity of virus entry providing a base for virus entry by endocytosis [92]. CD4 plays a prominent role in HIV entry and virus-cell interaction for T cells and for monocytes/macrophages. CD4 creates structural changes in viral proteins that allow HIV-1 to bind to a coreceptor expressed on the host cell, followed by insertion of a fusion peptide into the host cell and fusing with the membrane [55]. In turn, hMRC1-mediated uptake of HIV-1 by macrophages does not lead to productive infection in macrophages [86] but it facilitates virus transmission to T cells, which is important for virus dissemination and infection establishment This indicates that HIV-1 can also use the interaction with hMRC1 to its advantage [93]. Virus-induced receptor-mediated signalling can cause local actin rearrangement and facilitate phagocytosis, as was shown for cytomegalovirus [72]. HCMV binds to specific proteinaceous receptors—the β1 and β3 integrins—and to EGFR on the surface of monocytes, then triggers the activation of downstream signalling cascades [17]. The activation of both EGFR and integrins by HCMV is required for increased monocyte cellular motility through actin cytoskeletal rearrangement [17]. Actin rearrangement correlates with the translocation of viral capsids to the nucleus and infection, which is essential for the overall dissemination strategy of HCMV [94]. Numerous viruses can activate cells through different TLRs, including members of *Herpesviridae* (CMV, EBV) [82]. TLRs participate in the first line of defence against pathogens. They play a significant role in inflammation, immune cell regulation, survival and proliferation [95]. Although activation of TLRs might result in a protective antiviral immune response, it also may contribute to the pathology observed in infections caused by EBV [82]. Sensing of EBV by TLR2 results in increased secretion of proinflammatory cytokines, such as TNF-α, IL-1β, IL-6 and IL-8, which in turn contribute to the pathology observed in infections caused by EBV [82]. DC-SIGN and other C-type lectins act as pathogen recognition receptors that alert macrophages to take up and process pathogens for antigen presentation to T cells. Certain viruses such as HHV-8 can subvert this immune function by using DC-SIGN as a portal for immune dysfunction, resulting in the oncogenesis caused by HHV-8 infection [66]. Another example is the upregulation of xCT, which serves as a fusion-entry receptor for HHV-8 in infected macrophages; this receptor protects these cells from reactive nitrogen species-induced cell death [67].

In addition to the classic entry scenario that uses cell surface receptors and subsequent endocytosis, macropinocytosis, or membrane fusion for cell entry, some viruses infect macrophages through phagocytosis of apoptotic macrophages that were previously infected by that virus [96].

## 5. Productive Viral Infection

The fate of viruses and the cells affected by viral agents varies greatly depending upon the virus characteristics, although the fate can be grouped into 3 categories as follows: (1) persistence with productive viral replication; (2) persistence with a very limited time/amount of replication, or partial replication; and (3) restricted or short-term persistence with no/undetectable replication (cell here considered as a “random victim”). Monocytes/macrophages are the first subpopulation of immune cells that contact pathogens and can be infected and serve as a vehicle for virus dissemination; they are less likely to serve as a reservoir due to their naturally short life span and inability to support viral gene expression and replication [17]. Infected monocytes cross the blood-tissue barrier and disseminate viral particles as a specific parcels into the central nervous system, playing the role of the “Trojan horse,” which is common in HIV [1,2,11], HCV [38], HCMV [17] and Japanese encephalitis virus (JEV) [97]. Viruses need to overcome a number of hurdles to be able to successfully infect and replicate in monocyte/macrophage cells. According to an inner genetic program, after three days of circulation in blood vessels, monocytes must make a cell fate decision—either differentiate into tissue macrophages or default to biological programming and undergo apoptosis [17]. Here, viruses demonstrate their superb ability to modulate and navigate cells in order to escape degradation and replicate effectively in an organism. They evade apoptosis and prolong the cell’s life span via regulation of specific apoptotic pathways (PI3K and NFkB), that involves microRNAs [67,98] and modulation of the mitochondrial pathway [99]. They provoke changes in cell polarization, hide viral receptors from the cell surface and alter chemokine/cytokine expression in order to evade the immune response; they also affect cell motility to promote viral spread and dissemination in the body. Relevant data concerning viral persistence, cell responses and replication are summarized in Table 1 and are graphically presented in Figure 1 Several examples have confirmed these findings in detail.

An accumulation of evidence suggests that macrophages are not only random targets for HIV, they are also important and specialized viral reservoirs distributed throughout the body that store large amounts of unintegrated viral DNA in circular form in internal compartments [18]. HIV-1 infected macrophages were found in several body locations such as spleen, lung, heart, colon, brain and adipose tissue; this represents a major challenge for cure efforts due to the low drug delivery efficiency into these tissues [100,101]. Although CD4+ T cells are considered to be major permissive cells for HIV-1, the size of the virus reservoir is small. Approximately 1 infectious unit per million resting CD4+ T cells (1 IUPM) harbour the replication-competent proviral HIV-1 DNA [102]. Macrophages can also sustain a linear, steady amount of HIV-1 production, releasing replication-competent viral particles [103]. Attempts to assess the size of a viral reservoir in macrophages was recently carried out by Avalos C. R. et al. (2017) [100]. The authors applied a novel quantitative viral outgrowth assay in SIV-infected macaques that were ART-treated for 500 days and they revealed several interesting facts. First, even after antiretroviral therapy, 87% of suppressed animals contained latently infected brain macrophages, which were able to produce replication competent SIV. Second, unlike CD4+ T cells, the number of productively infected macrophages varied greatly across different tissues from the same macaque; this could be important for infection establishment and treatment strategy. Third, the highest levels of SIV production were found in spleen and brain macrophages (both microglial and perivascular) and was higher than the level of SIV production in CD4+ T cells, although these parameters could not be directly compared [100,104].

Macrophages are appealing to HIV-1 infection in many ways. They support productive replication and a life-long persistence as a latent reservoir that is provided by the cell machinery changes. HIV-1 adjusts macrophage for their existence by abolishing apoptosis and affecting several pathways that allow the virus to extend the period of persistence. HIV-1 infection of macrophages leads to overexpression of the Nef viral protein, which interacts with apoptosis signal regulating kinase-1. This result in the inhibition of Fas- and TNF receptor-mediated apoptosis and immune escape by preventing recognition by cytotoxic T lymphocytes [55,105,106]. Resistance to apoptosis also involves direct modulation of the mitochondrial pathway by regulating Bax pore induction [99]. Upon HIV-1 infection, macrophages increase their telomerase activity [107] and activate the expression of colony stimulating factor (M-CSF) protein [108]. After infection, macrophages actively secrete proinflammatory cytokines and chemokines that attract permissive cells within their vicinity, thereby transmitting virus to uninfected T cells, playing a crucial role in transmission and dissemination of HIV to other organs, including the brain [18]. The acute phase of HIV infection is characterized by a predominance of M1 macrophages expressing Th1 cytokines and chemokines (IFN-γ, IL-2, IL-12 and CCL3, CCL4, CCL5, respectively) and proinflammatory cytokines (TNF-α, IL-1β, IL-6 and IL-18). This inflammatory phenotype is characterized by a poor surface expression of CD4 and DC-SIGN, which are important receptors for HIV-1 binding [104]. The low abundance of specific receptors prevents superinfection in infected macrophages without restriction of viral replication [11]. However, at later stages of viral infection, there is a shift of macrophages to M2 due to the presence of IL-4 and IL-13, which favours the progression to AIDS [11]. Along with the above mentioned impairments, infected macrophages show phagocytosis impairments of apoptotic neutrophils during HIV infection, where the Nef protein plays a crucial role. The persistence of apoptotic neutrophils and their apoptotic bodies at the inflammatory site may maintain the inflammatory state through persistent stimulation of proinflammatory cytokines (TGFβ-1, prostaglandin E2 and platelet-activating factor) [109,110]. Macrophages also show anti-HIV-1 cellular restrictions such as the expression of *SAMHD1*, *APOBEC3A*, *APOBEC3G*, *tetherin*, *TRIM5-alpha* and *MX2* [17], suggesting the substantial importance of macrophages in HIV-1 pathogenesis. Monocyte differentiation into polarized macrophages and their dissemination into tissues are critical for the establishment of HIV-1 infection. First, resident tissue macrophages remain in tissues long term, with a capability of self-renewal upon cell machinery changes; second, macrophages are relatively resistant to the cytopathic effects of HIV infection compared to CD4+ T cells. Together, these peculiar qualities provide a basis for the formation of a stable viral reservoir, which is recognized as a major barrier to curing HIV-1 infections [104].

There is a wide range of disease pathologies seen in several organ sites associated with human cytomegalovirus infection. HCMV is involved in chronic inflammation and the development of cardiovascular diseases and some types of cancers in immunocompetent individuals [111,112]. HCMV has the potential to contribute to tumour progression by oncomodulation through the production of viral proteins, affecting cellular differentiation, gene expression, DNA replication and cell cycle progression [113]. The virus leads to immunosuppression, which may further lead to immunotolerance against the growing tumour [113]. HCMV-induced pathologies are mediated predominately by infected monocytes, which serves as a permissive system and are a long-term reservoir for the virus. Productive virus release of HCMV from monocytes/macrophages is detectable up to 16 weeks after infection [17]. This virus overcomes a number of biological hurdles in the monocytes to provide support for viral gene expression and replication. Monocytes become permissive only upon their differentiation into macrophages, which is driven by the virus, resulting in progeny virions capable of infecting the necessary surrounding cell types. Monocyte-to-macrophage differentiation is partially mediated by caspase-3 activation [17]. It was shown that HCMV could specifically regulate the polarization of infected monocytes/macrophages to achieve an effective balance between proinflammatory and anti-inflammatory signals. This regulation may establish a cellular environment that is conducive for the dissemination and persistence of HCMV (the “Goldilocks” phenomenon) [17]. During latency, HCMV modulates cytokine/chemokine secretion for the biased recruitment of immune cells to propagate latency in the host. Stevenson E. et al. (2014) [17] hypothesized a very specific role of HCMV in monocyte/macrophages modulation. According to this hypothesis, viruses promote a “finely-tuned” cell type, where monocytes/macrophages exist somewhere along the M1/M2 continuum that is needed for viral spread, replication and persistence. HCMV drives the simultaneous expression of M1- (IL-6, TNF-α, CD86) and M2-associated molecules (IL-10 and CD163). HCMV likely employs M1-associated markers and chemokines to promote the proinflammatory activation of infected monocytes/macrophages. This ensures elevated cellular motility, migration and monocyte recruitment, which has also been observed for infected tumour cells [113]. At the same time, HCMV use M2-associated macrophage markers and chemokines to silence the tissue-damaging effects of the pro-inflammatory response and any potential anti-viral responses [17]. HCMV belongs to the agents capable of long-lasting infection due to cell apoptosis decoy. Thus, after infection, cytomegalovirus induces activation of *EGFR* and the β1 and β3 integrins on the surface of monocytes, leading to the prolonged survival of infected cells by the prolonged expression of *Mcl-1* [98,114].

The long persistence in liver macrophages was observed for a member of *Flaviviridae* family—the hepatitis C virus, albeit in small quantities. It was detected in cells up to 9 years after therapy [37]. Chronic HCV infection can lead to advanced liver fibrosis, cirrhosis and hepatocellular carcinoma and constitutes a significant health burden worldwide [115]. Despite the tropism to hepatocytes, monocytes/macrophages also play an important role in HCV replication in vivo, showing productive replication of a virus in a relatively nonspecific manner as long as macrophages survive [116]. Flaviviruses successfully replicate their genome and can evade and/or subvert the macrophage response to favour survival and replication due to elevated *TNF* expression, along with the increased expression of *NOS2* and the antiviral (but immune-suppressive) enzyme indolamine-2-3 dioxygenase production [40]. In addition to hepatocytes, microglial cells of HCV-infected individuals have been shown to be virus-positive, demonstrating significantly higher levels of proinflammatory cytokines IL-1a, IL-1b, TNFa, IL-12 and IL-18 and increased transcription of chemokines IL-8, IL-16 and IP-10 [38]. Some authors speculate that the release of proinflammatory cytokines and neurotoxins such as NO and HCV viral proteins upon infection could potentially induce changes in brain function, leading to neurocognitive dysfunction and depression [38].

Members of *Togaviridae*, the Chikungunya alphavirus and the Ross River virus, have been shown to persist in macrophages for a long time. CHIKV persist in synovial tissue after 18 months in patient after infection [50], with the productive replication of virus in synovial macrophages [20]. The Ross River virus showed high level of replication in macrophages shortly after infection but viral antigens were also detectable by IFA analysis after 170 days in an in vitro model, indicating that the virus had not been completely cleared from the cells [52]. Despite the induced apoptosis in many cell types, CHIKV appears to launch a specific innate immune response in infected cells through the increased levels of TNFα, controlled by NFkB activation [49]. Moreover, this virus utilizes a high cell mortality for its dissemination into apoptotic blebs [20].

There are several examples of persistence that are very limited in the time or amount replication. It is known that lytic productive replication leads to high mortality of permissive cells but not in the case of macrophages. For example, macrophages carrying virus particles can survive due to the limited replication of virus, as was demonstrated for Frog virus 3 [25]. This phenomenon provides additional time for the dissemination in organism and to infect other cell populations in the vicinity. Among the viruses with short replication times in macrophages are the Theiler’s murine encephalomyelitis virus (TMEV) and the respiratory syncytial virus (RSV). Experiments in vitro using different cell lines have demonstrated the upregulation of IL-10 and the downregulation of IFN-α, INF-β and IFN-g, which may contribute to the TMEV persistence. Upregulation of these mediators, as well as B-lymphocyte chemoattractant (BLC) and granulocyte colony-stimulating factor (G-CSF), were observed in macrophages after infection and may contribute to the acceleration of TMEV-induced demyelination resembling multiple sclerosis [46]. Information regarding cell machinery modifications upon TMEV infection is contradictory. There are alterations in the immune response of those cells that contribute to chronic inflammatory responses from macrophages and changes that allow infected macrophages to escape from immune response [117,118]. Productive RSV infection was found in isolated human alveolar macrophages in vitro for at least 25 days, suggesting that macrophages may be important targets for RSV during acute infection [26]. RSV-infected cells showed lack of autocrine response to the constitutively produced IFN-β by inhibition of *STAT1* phosphorylation. This may prevent the transcription of antiviral genes and consequently allow the maintenance of persistent RSV infection [27].In addition to a group of viruses that can replicate their genome and produce infectious particles in monocyte/macrophage cells, there are a set that carry out a latent infection, exploiting cells only as a potential reservoir for viral storage.

## 6. Latent Viral Infection and Inflammation

Some infection agents use macrophages to establish a latent infection. Viral latency characterized by the ability of a pathogenic virus to lie dormant within infected cells. It is a state of reversibly non-productive infection of cells and provides an important mechanism for viral persistence and escape from immune recognition and drug pressure [104]. It could contribute to several pathologies by promoting chronic inflammation and a subsequent predisposition to cognitive impairments, inflammatory demyelinating disease and multiple sclerosis, precancerous lesions and cancer [12]. Human herpesvirus 8 (HHV-8) infection correlates with an elevated risk of prostate cancer development. HHV-8 was shown to establish a chronic latent infection in epithelial cells and macrophages, which contributed to increased macrophage infiltration in tissue that serve as a cofactor for prostate cancer development in Tobagonian males [68]. Other viruses, such as retroviruses, flaviviruses, alphaviruses, picornaviruses and rhabdoviruses, are commonly associated with skeletal muscle infection and inflammation, leading to either direct infection of myofibers or infiltrating inflammatory macrophages [48]. Although macrophages are not the major reservoirs of varicella zoster virus (VZV), infection causes an intense inflammatory response during the reactivation of VZV, leading to widespread necrosis of glial cells and neurons [70]. Clinically, herpes zoster is associated with severe, acute pain and frequently, prolonged severe pain or post herpetic neuralgia. It often requires follow-up medical care for months or even years after the initial attack [70].

A member of the *Flaviviridae* family, Zika virus, was identified in 1947 but initially garnered little interest from scientists. Today, it attracts much attention within the scientific community given the recent evidence of linkages to microcephaly in new-borns, central nervous system abnormalities, foetal growth restriction, maternal Guillain–Barré symptoms and its rapid spread around the globe [3]. Human placental macrophages, called Hofbauer cells, play a prominent role in virus dissemination. Zika virus replicates in macrophages for at least 96 hours after infection in vitro, which lead to little induction of pro-inflammatory cytokines and chemokines and antiviral gene expression, with minimal cell death [41]. It results in a weaker immune response that prolongs the virus infection and persistence in body tissues [119] Infected macrophages showed strong activation of migration inhibitory factor (MIF), which leads to a higher migration ability of infected cells and can boost virus ability to cross the placental barrier and promote its spread in the body [119].

Several types of viruses utilize macrophages as a potential reservoir for viral storage in the absence of replication, as was demonstrated for vesicular stomatitis virus [32]. It was shown that macrophages are the major target cells of Maedi-visna virus despite very limited replication in the animal host [57]. HHV-6 infection is typically non-productive in macrophages; nevertheless, it induces severe functional abnormalities, including the selective suppression of IL-12, a critical cytokine in the generation of M1-polarized antiviral immune responses [63]. An additional mechanism of cell transformation utilized by HHV-6 is immunomodulation, which includes alterations in the cell surface receptor expression that can facilitate its own spread and persistence [63].

Therefore, it has to be assumed that viruses manipulate the cell biology of monocytes/macrophages in order to utilize them as a repository for dissemination, to promote long-term persistence within infected tissues and for enabling virus replication under favourable conditions.

## 7. Macrophages as a Target for Therapeutic Intervention

Monocytes/macrophages play a critical role in several virus-mediated diseases and represent a major hurdle for treatment. Therapeutic targeting of macrophages is challenging, as these cells reside broadly dispersed in nearly all tissues, including those that are difficult to access with drugs such as the CNS and adipose tissue. Nevertheless, several attempts have been undertaken.HIV-1 persists in CD4+ T cells and macrophages and cannot be eliminated by antiretroviral therapy [106]. There are several therapeutic agents available on market; however, for macrophages, protease inhibitors (PIs) are currently the only approved drugs. One of the hurdles to clinical implementation is the pharmacokinetics of a drug. When targeting macrophages, approximately 15- to 89-fold higher concentrations of PIs are required compared with CD4+ T cells [120]. Despite the failure of macrophage-HIV eradication, another innovative idea has been successfully implemented. Liao H.K. et al. (2015) [121] showed a significant reduction of HIV-1 expression by using a multiplexed CRISPR/Cas9 system. The result was also confirmed in primary CD4+ T cells [122].

When activated by HCV, macrophages play a critical role in hepatic inflammation and fibrosis progression [123]. The approved direct-acting antiviral (DAA) therapy for HCV patients aims for viral clearance, mainly in hepatocytes. However, recent data show a positive effect of the treatment on myeloid cells. A sustained virological response, as assessed by the kinetics of soluble CD163 in the serum of patients, was achieved for the clearance of the HCV virus, with consequent mitigation of inflammation, confirming the relevance of monocyte/macrophage populations in HCV-mediated liver pathologies [123]. JEV and West Nile virus are the leading cause of acute viral meningoencephalitis worldwide [124]. Recently, a pharmacologic method of microglia depletion (PLX5622, Plexxikon Inc., Berkeley, CA, USA, an inhibitor of colony-stimulating factor 1 receptor) has been applied experimentally to evaluate macrophage involvement in viral clearance and CNS injury as part of the neuroinflammatory process [125]. It is known that microglial cells are a significant target for the Japanese encephalitis virus [125]. Seitz S. et al. (2018) [125] showed that PLX5622 treatment lead to a dramatically increased disease severity and increased virus titres and mortality following Flavivirus infection. The authors speculate that microglia could have an additional protective function that is critical in controlling viral infection [125]. These data demonstrate the crucial role of macrophages in virus establishment and clearance and should be taken into consideration when targeting macrophages in therapeutic interventions. Apart from antiviral therapy, nonsteroidal anti-inflammatory drugs are used for virus-induced pathologies; this has been applied for the Ross River virus and other viral arthritides [126]. RRV causes arthralgia and/or arthritis, with many patients experiencing myalgia and fatigue and some experiencing fever and rash [127]. Macrophages are central players in the onset of viral arthropathies, expressing a spectrum of cytokines and chemokines upon infection. Rulli N.E. et al. (2009) [126] demonstrated that bindarit-based treatment (an inhibitor of chemokine synthesis) reduced macrophage infiltration into the muscles and joints, which led to reduced *TNFα* and *NOS2* expression; this resulted in reduced tissue damage and a significant amelioration of disease symptoms.

## 8. Conclusions

Accumulating evidence demonstrates that despite being a suboptimal replication system, monocytes/macrophages cannot escape viral attack. Pathogens attack a variety of cells and utilize diverse bypass mechanisms to enter, change cell machinery and impact the fate of cellular decisions in favour of their replication and propagation.

Several questions remain unclear and have potential for future investigations. What is the longevity of infected macrophages in tissues? How big are the size of macrophage reservoirs in tissues upon different infections and what impacts on disease progression and therapy could this have? The role of the microbiome in infection and its changes upon viral infection has attracted increasing scientific attention. Although this field is unexplored for the majority of viruses, pilot data has obtained, for example, for HIV-1 and HCV infections [128,129,130,131,132]. Several publications have described a decrease of gut microbial alpha-diversity, that inversely correlates with monocyte activation upon microbial translocation in HIV-1-infected patients compared to healthy controls [128,131]; gut dysbiosis, accompanied by systemic inflammation and endotoxemia in HCV-infected individuals [129]. But how do infected macrophages interact with the microbiota to influence disease onset and progression? This information could guide further development of effective therapies, with the aim to reduce microbial translocation and misbalance, diminish subsequent immune activation and reduce morbidity and mortality in virus-driven diseases. These small populations of infected monocytes/macrophages have the potential to be milestones in the understanding of virus-induced diseases and their complications, playing a crucial role in inflammation and possibly in systemic pathology.

## Figures and Tables

**Table 1:** Viruses affecting monocyte/macrophage cells.

	Virus	Family	Genome	Host	Disease	Model	Type of Cells	Virus Persistence and Survival in Mφ	Reaction of Mφ	Reference
1	Respiratory syncytial virus (RSV)	*Paramyxoviridae*	(−) ssRNA	Human	Bronchiolitis and pneumonia, severe acute lower-respiratory tract disease in children	-In vitro model of persistently RSV-infected Mφ-like cell line P388D1 (MφP) -RAW264.7 Mφ -Human tissue Mφ	-Murine Mφ-cell line -Human alveolar Mφ	-RSV persist in in vitro model for over 87 passages. -Alveolar Mφ support a productive RSV infection in vitro at least for 25 days. -Lack of response of infected Mφ to the IFN-beta.	-Infected Mφ produces high level of proinflammatory cytokines (class II HLA-DR, IL-1ß and TNFα—immunofluorescent staining). -Reduced cytotoxic effect in MφP cells (activation of caspase-9 along with impairment of caspase-8 activity).	[26,27,28]
2	Influenza virus	*Orthomyxoviridae*	(−) ssRNA	Human	Influenza	-Autopsies -In vitro model, BALB/c mice -Human primary Mφ	-Mouse lung Mφ -Human primary Mφ	-Low virulence persistence of influenza virus in the alveolar Mφ. -Productive replication of H5N1 virus in alveolar Mφ.	-Infected Mφ produces high level IL-1ß, IL-6, TNFα (flow cytometry). -Mφ demonstrated no cytopathic changes (visual examination of monolayers).	[29,30,31]
3	Vesicular stomatitis virus (VSV)	*Rhabdoviridae*	(−) ssRNA	Insects, cattle, horses, pigs (zoonotic virus)	Flu-like illness in infected humans	BALB/c mice	Tissue Mφ (lymph nodes, lungs, spleens, liver, muscle)	-Mφ are not the major reservoirs of VSV gRNA at late times (>60 days). -No replication in Mφ.	NS	[32]
4	Porcine reproductive and respiratory syndrome virus (PRRSV)	*Arteriviridae*	(+) ssRNA	Pig	Respiratory syndrome	Pig	Porcine alveolar Mφ	Productive replication of virus in alveolar Mφ in in vivo model.	In vitro infected Mφ are protected against complement-mediated cell lysis.	[23]
5	Feline coronavirus (FCoV)	*Coronaviridae*	(+) ssRNA	Cats	Infectious peritonitis	Specific-pathogen-free cats	Tissue Mφ	Virus persists in tissue Mφ (mostly in colon) up to 80 days after inoculation.	NS	[33]
6	Mouse hepatitis virus (MHV)	*Coronaviridae*	(+) ssRNA	Mouse	Model of multiple sclerosis	Mouse	Mouse peritoneal Mφ	-Mφ disseminate virus into CNS. -Mouse peritoneal Mφ are permissive for virus. Virus persists in the cells up to 8 months.	Infected Mφ express CCR1, CCR2 and CCR5 that lead to recruitment of Mφ into the CNS.	[34,35]
7	Classical swine fever virus (CSFV)	*Flaviviridae*	(+) ssRNA	Swine	Lethal fever	Pig tissue monocytes and Mφ	Tissue monocytes and Mφ	Productive replication of virus in alveolar Mφ and monocytes.	NS	[36]
8	Hepatitis C virus (HCV)	*Flaviviridae*	(+) ssRNA	Human	Hepatitis C	-Patients -Cell culture (THP-1)/tissue Mφ	-PBMC, Mφ culture (THP-1), microglial Mφ (CD68+ and CD45+)	-HCV persists in liver Mφ and lymphocytes for up to 9 years. -Productive replication of virus in a relatively non-specific manner in Mφ.	Infected Mφ/microglial cells express higher level of IL-1α, TNFα, IL-1β, IL-12, IL-18 (PCR analysis).	[37,38]
9	Japanese encephalitis virus (JEV), West Nile virus (WNV), Dengue virus (DENV)	*Flaviviridae*	(+) ssRNA	Human	Neurotropic, CNS	-Raw264.7 cells -BALB/c mice -Macaques	-Murine monocyte-derived Mφ (MDM) -Perivascular Mφ	-Productive JEV and WNV replication in murine and human Mφ. -Intracellular persistence of virus in Mφ. -Flaviviruses evade and/or subvert the Mφ response to favour survival and replication.	-Infected Mφ produce TNF-α, IL-6, IFN-α and CCL2, inducible nitric oxide synthase (iNOS) and nitrotyrosine (NT) in response to JEV in vitro (immunofluorescent staining, IFN bioassay, Cytometric Bead Array). -Both human and rodent microglia produce CCL2, CXCL9 and CXCL10 upon JEV exposure (flow cytometry).	[22,39,40]
10	Zika virus (ZIKV)	*Flaviviridae*	(+) ssRNA	Human	Foetal brain abnormalities and microcephaly, Guillain–Barré syndrome	Primary human placental Mφ	Human placental Mφ (Hofbauer cells, HC)	-Hofbauer cells are permissive to productive ZIKV infection.	Infected HCs produce high level of IFNα, IL-6, chemokines MCP-1 and IP-10 (flow cytometry).	[41]
11	Coxsackieviruses (CVB4, CVB3)	*Picornaviridae*	(+) ssRNA	Human	-Type 1 diabetes; -Myocarditis	-Human MDM; -Mouse	-MDM -Pancreas Mφ -Myocardial Mφ (activation of JAK1-STAT1 and JAK3-STAT6 pathways)	-CVB4 replicates and persist in MDM and tissue Mφ.	-CVB4-infected Mφ produce high levels of pro-inflammatory cytokines (IL-6 and TNFα—ELISA) in both M-CSF MDM and GM-CSF MDM cultures. -Virus infected pancreas Mφ showed M1 polarization (Ly-6C+/CD115+—flow cytometry). -Mφ polarization depends on gender (M1 phenotype detected in males and M2a phenotype in females).	[42,43,44]
12	Encephalomyocarditis virus (EMCV)	*Picornaviridae*	(+) ssRNA	Rodents, pigs	-Lethal acute myocarditis, fatal illness in primates and captive wild animals	-Mouse tissue Mφ -RAW264.7, naive mouse Mφ	-Tissue Mφ (brain, heart, pancreas, kidney, Peyer’s patches, spleen, lung and thymus)	-Virus persists in the thymus Mφ up to 62 days post infection. -Productive viral replication in Mφ.	EMCV activates pro-inflammatory signalling in Mφ within minutes during virus infection and type I IFNs response afterwards.	[24,45]
13	Theiler’s murine encephalomyelitis virus (TMEV)	*Picornaviridae*	(+) ssRNA	Mouse	Skeletal muscle infection and inflammation, encephalomyelitis and multiple sclerosis, epilepsy	-J774.1 Mφ -RAW264.7 Mφ cell line -C57BL/6 (B6) mouse -Primary peritoneal Mφ	Mφ cell lines, tissue Mφ	-TMEV persists in Mφ during the chronic demyelinating phase. -Productive replication of TMEV in Mφ.	-Infected Mφ in wild type animals showed M1 polarization (CD45+CD11b+Ly6c+) while muscle-infiltrating Mφ displayed an immature phenotype in SHP-1-deficient mice (flow cytometry). -Infected peritoneal Mφ produce high level of IFNα and TNFα (ELISA). -TMEV replication cause restricted induced apoptosis of Mφ.	[16,46,47,48]
14	Chikungunya alphavirus (CHIKV)	*Togaviridae*	(+) ssRNA	Human	Articular disease/ myalgia	-Cell line -Patients -Immunocompetent cynomolgus macaques	-RAW264.7 Mφ -Perivascular synovial Mφ	-Productive replication of CHIKV in RAW264.7 Mφ but in in vivo—low viral replication and release of non-infectious viral particles. -Virus persists in the cells after 18 months of chronic disease. -Mφ are the main cellular reservoirs during the late stages of CHIKV infection in vivo.	No induced apoptosis in infected RAW264.7 cells.	[19,49,50]
15	Sindbis virus (SINV), Mayaro virus (MAYV), O’nyong-nyong virus (ONNV) and Barmah Forest virus (BFV), Ross River virus (RRV), CHIKV	*Togaviridae*	(+) ssRNA	Mosquitoes, marsupials, humans	Articular disease/myalgia	-Patients -RAW 264.7 Mφ	-RAW264.7 Mφ -Perivascular synovial Mφ	-Productive viral gene expression in synovial Mφ. -RRV persist in RAW 264.7 Mφ up to 170 days in vitro.	-Infected Mφ displayed M1 polarization (CD68+) in vivo. -CHIKV infection cause induced apoptosis in vivo leading to viral dissemination into apoptotic blebs. -CHIKV-infected RAW264.7 Mφ showed high production of TNF-α, IL-6 and GM-CSF (QPCR). -RRV-infected Mφ in vitro displayed restricted cytopathic effects.	[20,51,52,53]
16	Avian oncoviruses	*Retroviridae*	ssRNA-RT	Many species	Cancer	Chicken	Tissue Mφ, MDM	-Avian leukosis viruses persist in Mφ of peripheral blood up to about 3 years. -Avian sarcoma viruses were never found in Mφ.	NS	[14]
17	Murine leukaemia viruses (MuLVs)	*Retroviridae*	ssRNA-RT	Mouse	A model for non-inflammatory degeneration of the central nervous system	BALB/c and C3H mice	Tissue Mφ	Virus infects Mφ/microglia and persists during later stages (8 weeks after infection).	NS	[15]
18	Ovine lentivirus OvLV	*Retroviridae*	ssRNA-RT	Sheep	Encephalitis and chronic pneumonitis	Lamb	Tissue Mφ	OvLV variants persist in alveolar Mφ.	NS	[54]
19	Human immunodeficiency virus (HIV), Simian immunodeficiency virus (SIV)	*Retroviridae*	ssRNA-RT	Human	Immune deficiency syndrome (AID), cancer	Human (U937, THP-1) and mouse cell lines, human and monkey, macaque tissue Mφ	MDM, monocytes, tissue Mφ	-CD14+CD16+ monocytes are permissive to productive infection. -Mφ serves as a major reservoir for HIV. -Infected Mφ escape immune response. -Infected Mφ showed impaired phagocytic activity. -Mφ dissiminate HIV to CD4+ T cells and central nervous system (“Trojan horse hypothesis”).	-Infected Mφ showed M1 polarization during early stages of infection (with high production of IFN-γ, IL-2, IL-12—ELISA). There is a shift of M1 to M2 at later stages of infection (with high production of IL-4, TGF-β and IL-10—ELISA). -HIV-1 infection enhances the survival of Mφ by upregulating antiapoptotic genes through different pathways (activation of NF-kB and PI3K signalling, delay of TNF-induced apoptosis; modulation of mitochondrial pathways; increase telomerase activity).	[18,55,56]
20	Maedi-visna (MVV)	*Retroviridae*	ssRNA-RT	Sheep	Fatal lymphoproliferative disease	Sheep	Bone marrow monocytes, PBMC	Limited virus replication in bone marrow monocytes.	NS	[57]
21	African swine fever virus (ASFV)	*Asfarviridae*	dsDNA	Pig, warthogs, bushpigs, soft ticks	Lethal haemorrhagic fever	-Pig -Porcine alveolar Mφ	-Cell culture derived from bone marrow, PBMC -Alveolar and bone marrow cells	-Virus persists in tissue Mφ. -Moderate virus replication continued for at least 3 months in alveolar and bone marrow Mφs.	-Virus caused cytotoxic effect within 2–3 days in monocytes but not in Mφ (visual examination of monolayers). -Virus leads to full morphological differentiation of Mφ (visual examination of cell morphology).	[13,58]
22	Bovine herpesvirus-4 (BHV-4)	*Herpesviridae*	dsDNA	Cattle, rabbits	Endometritis, vulvovaginitis and mastitis	Bovine Mφ cell line (BOMAC).	Cell culture	-Virus cause cell death of the majority of BOMAC cells and persists in surviving cells.	NS	[59]
23	Cytomegalovirus (CMV)	*Herpesviridae*	dsDNA	Human	Chronical inflammation, cardiovascular diseases, some types of cancers	-Murine cytomegalovirus model (MCMV), MDM/Allo-MDM	-Human monocytes -MDM	-Productive replication of CMV in human Mφ up to 16 weeks but not monocytes. -Monocytes disseminate virus in organism.	-HCMV induces specific phenotype within M1/M2 continuum (skewed towards M1). Simultaneous expression of M1-associated molecules (IL-6, TNF-α, CD86) and M2-associated molecules (IL-10 and CD163) by infected Mφ. Data analysed using PCR, flow cytometry (M1 cells were CD68+, M2—CD163+), microarray analysis for more than 2000 genes. -Decoy of induced apoptosis of infected monocytes due to prolonged expression of the anti-apoptotic molecule, Mcl-1. -Infected cells escape the cellular antiviral pro-apoptotic response due to specific cytokine/chemokine expression (the “Goldilocks” phenomenon). -Infected cells utilize EGFR receptor and integrins.	Review [17,60]
24	Epstein-Barr virus infection (EBV)	*Herpesviridae*	dsDNA	Human	Inflammation, some types of cancers	Human cancer tissues, human smears, rhesus macaques, Mφ culture (RAW 264.7 cells), Balb/c and IL-10KO mice.	MDM, tissue Mφ, submucosal monocytes, tumour-associated Mφ (TAMs)	-EBV replicates in Mφ. -Monocytes disseminate virus in organism.	-Infected Mφ produce high level of IL-8, MCP-1 due to TLR9 and TLR-2 activation (ELISA). -Monocytes produce high level of IFNα in response to EBV (ELISA). -IL-10-dependent M2 polarization of infected TAMs (ELISA).	A book [61], review [62]
25	Human herpesvirus 6 (HHV-6)	*Herpesviridae*	dsDNA	Human	Multiple sclerosis	Human	-PBMC	-Latent persistence of HHV-6 in Mφs for more than 1 month.	Selective downregulation of IL-12 in infected Mφ (ELISA), which is not dependent upon productive viral infection.	[63,64,65]
26	Kaposi’s sarcoma-associated herpesvirus KSHV (HHV-8)	*Herpesviridae*	dsDNA	Human	Cancer	-Tumour microenvironment, cell culture. -MDM -Prostate cancer samples	Tissue Mφ, RAW 264.7 cells	-HHV-8 led to production of viral proteins in intralesional Mφ, with little production of viral DNA. -Virus persists in a latent form in Mφ/monocytes. -Lytic gene expression in Mφ in prostate stroma.	KSHV miRNAs protect Mφ from cell death through the upregulation of xCT.	[66,67,68]
27	Murine herpesvirus 72 (MHV-72)	*Herpesviridae*	dsDNA	Mouse	Acute infection of lung epithelial cells	Balb/c mice	Lung mononuclear cells	Virus persists in alveolar and peritoneal lung mononuclear cells and Mφ of peripheral blood up to 8 months.	NS	[69]
	Varicella-zoster virus (VZV), simian varicella virus (SVV)	*Herpesviridae*	dsDNA	Human, nonhuman primates	Varicella-zoster, “multiple sclerosis-like” pathology	-Human ganglia. -Rhesus macaques.	Alveolar Mφ	-SVV IE63 proteins are present in Mφ in lymph nodes after SVV reactivation in monkeys. -SVV infects alveolar Mφ and transmit virus to T cells.	SVV-infected Mφs were CD163+ (immunofluorescence analysis) after virus reactivation but not during latency.	[70,71]
29	Frog virus 3 (FV3)	*Iridoviridae*	dsDNA	Amphibian species	Acute systemic FV3 infection	*Xenopus laevis*	Peritoneal Mφ	FV3 persist in peritoneal Mφ in vitro.	No cytopathic effect on infected Mφ.	[25]

**Table 2:** Virus-host cell interactions.

	Virus	Entry Type	Receptor(s) Used for Viral Entry/Attachment	Virus Fate	Reference
1	Respiratory syncytial virus	Macropinosome formation	Nucleolin, heparan sulphate proteoglycans	Replication	[75,81]
2	Influenza virus	Endocytosis, Phagocytosis	Sialic acid sugars	Replication	[76]
3	Vesicular stomatitis virus	endocytosis	Phosphatidylserine	No replication	[32,79]
4	Mouse hepatitis virus	Phagocytosis	* Sialic acid sugars and glycolipids N- * acetilneuraminic acid receptor	No replication	[34,77]
5	Japanese encephalitis virus, West Nile virus, Dengue virus	Phagocytosis	* DC-SIGN or * DC-SIGNR TLR-2, TLR-3 and TLR-7, RIG-I	Replication	[39,40]
6	Coxsackieviruses	Macropinocytosis	* CAR and IgG Fc fraction receptors (FcγRII and FcγRIII), occludin	Replication	[42,73,84]
7	Theiler’s murine encephalomyelitis virus	Endocytosis	Sialic acid sugars	Replication	[46,78]
8	Human immunodeficiency virus, Simian immunodeficiency virus	Endocytosis, macropinocytosis	Human mannose receptor C-type 1 * CD4 and a * coreceptors CXCR4 or CCR5, heparan sulphate proteoglycans	Replication	[11,85,86]
9	Cytomegalovirus	Endocytosis	Heparin sulphate proteoglycans following by the binding to the β1 and β3 integrins, EGFR, TLR2, TLR3 and TLR9 (murine CMV)	Replication	[17,82]
10	Epstein-Barr virus infection	Endocytosis	* CR2 or CD21, TLR2 and TLR3	Replication	[82]
11	Human herpesvirus 6	Endocytosis	* CD46	Non-productive infection	[63,87]
12	Kaposi’s sarcoma-associated herpesvirus	* Macropinosome membrane fusion	xCT, DC-SIGN, * surface heparan sulphate, * integrin α3β1 (CD49c/29)?	Replication	[66,67,68,69,70,71,72,73,74,75,76,77,78,79,80,81,82,83,84,85,86,87,88]
13	Varicella-zoster virus, Simian varicella virus	Fusion with the plasma membrane or endocytosis	* Mannose-6-phosphate receptor, myelin-associated glycoprotein	not clear	[71,89]

* Data nonspecific for Mo/Mφ.

## Abbreviations

Mo	Monocyte
Mφ	Macrophage
MDM	Monocyte-derived macrophages
HC	Hofbauer cells
HIV	Human immunodeficiency virus
HCMV	Human cytomegalovirus
RSV	Respiratory syncytial virus
VSV	Vesicular stomatitis virus
dsDNA	Double stranded DNA
ssRNA	Single stranded RNA
CSFV	Classical swine fever virus
EMCV	Encephalomyocarditis virus
MHV	Mouse hepatitis virus
ZIKV	Zika virus
MVV	Maedi-visna virus
EBV	Epstein-Barr virus
KSHV	Kaposi’s sarcoma-associated herpesvirus
HHV-6	Human herpesvirus 6
VZV	Simian varicella-zoster virus
JEV	Japanese encephalitis virus
AIDS	Acquired immune deficiency syndrome
CHIKV	Chikungunya alphavirus
TMEV	Theiler’s murine encephalomyelitis virus
